# The Right Gastroepiploic Artery as a Potential Organ at Risk in Neoadjuvant Chemoradiation for Esophageal and Gastroesophageal Cancers

**DOI:** 10.7759/cureus.61342

**Published:** 2024-05-30

**Authors:** Adham Hijab, Yonina Tova, Shlomi Alani

**Affiliations:** 1 Radiotherapy, Ziv Medical Centre, Safad, ISR

**Keywords:** gastroesophageal cancer, conduit failure, anastomotic leak, esophageal cancer, neoadjuvant chemoradiation

## Abstract

Background: Preoperative chemoradiation is a standard of care for esophageal and gastroesophageal cancer. A gastric conduit is usually used for anastomosis with the right gastroepiploic artery (RGEA) being the sole arterial supply to the gastric remnant after such surgeries. Hence, lowering the radiation dose to this vessel may lower the risks of postoperative complications related to poor vasculature. Herein, we report our experience in contouring and replanning cases of distal esophageal/gastroesophageal carcinomas so that the radiation doses to the RGEA could be minimized.

Materials and methods: Radiation plans of patients with lower esophageal/gastroesophageal carcinomas were retrieved from our database. Identification and delineation of the RGEA was done and replanning was performed with the aim to keep the maximal and mean doses as well as the V10Gy and V20Gy of the RGEA as low as possible without compromising target volume coverage.

Results: We achieved significant dose reductions in most of the dosimteric parameters in our selected cases without compromising target coverage.

Conclusion: Lowering the dose to the RGEA, a potential organ-at-risk that may impact the postoperative course after neoadjuvant chemoradiation, is feasible.

## Introduction

Esophageal cancer is the eighth most common cancer and the sixth most common cause of cancer-related death worldwide [1]. Neoadjuvant chemoradiation (NACRT) is a standard of care for patients with resectable esophageal/gastroesophageal junction (GEJ) carcinoma, as it improves R0 resection (microscopically negative-margins after resection) rates, loco-regional relapse, and overall survival [2,3].

Despite the variability of the esophagectomy techniques that could be utilized, a gastric conduit reconstruction is always preferred. The right gastroepiploic artery (RGEA), often becomes the sole arterial supply to the gastric tube used for anastomosis [4]. In this sense, preserving adequate blood supply via the RGEA could lower postoperative complications and ensure improved healing of the anastomosis. Indeed, the reported postoperative complications that could be attributed to anastomotic failure, such as anastomotic leakage or conduit ischemia, are not negligible [5-7]. During NACRT to lower esophageal/GEJ carcinomas, the radiation dose to the celiac trunk branches (including the RGEA) is potentially significant, and this may lead to radiation-induced endothelial damage [8]. Moreover, concomitant chemotherapy given during NACRT could also potentiate endothelial injury [9]. Impaired endothelial function can eventually lead to inadequate blood supply with subsequent end-organ ischemia [10].

In this work, we sought to evaluate the dosimetry of the RGEA in patients with lower esophageal/GEJ carcinomas, and to determine whether a significant dose reduction was achievable when replanning was performed.

## Materials and methods

Patients with lower esophageal/GEJ carcinomas who were treated at our department between 2019 and 2023 with NACRT, followed by surgery were eligible to be included in the study. All patients were instructed to fast four hours prior to planning CT. Patients drank 50 cm^3^ of oral contrast and were simulated with a deep-inspiration breath-hold computed tomography (CT) using a 32-slice scanner (Toshiba Aquilion LB, Toshiba Medical Systems Europe, Zoetermeer, the Netherlands). CT scans were contoured and planned using Varian Eclipse version 17.0.0.0 (Varian Medical System, Palo Alto, CA, USA), with a 2.5 mm grid size.

**Figure 1:**
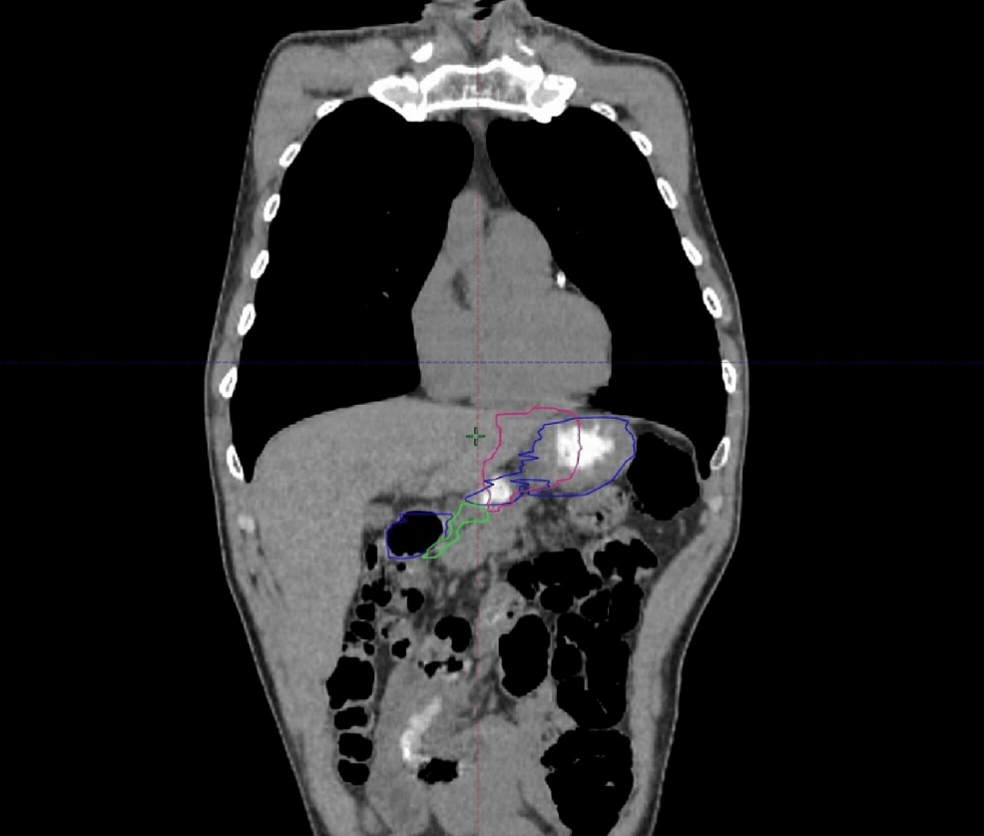
CT image of the patient (coronal view) A coronal representative image of the RGEA (green structure) running along the greater curvature of the stomach (blue) in its right aspect. Note the proximity between the RGEA and the PTV (pink) as in this patient the celiac nodes were covered in the target volume.

All patients received NACRT based on CROSS protocol [2]. Namely, patients received 41.4 Gy in 23 fractions concurrently with weekly chemotherapy, which constituted carboplatin with area under curve (AUC) of 2 and paclitaxel at a dose of 50 mg per square meter of body-surface area. The clinical target volume (CTV) encompassed the primary gross tumor volume (GTVp) with a 4 cm cranio-caudal expansion (with a 2 cm expansion into the uninvolved stomach in GEJ tumors) and a 1 cm radial expansion. The celiac and gastrohepatic nodes were included in the CTV. A 5 mm expansion around gross nodal volume (GTVn) was added for CTV generation. The planning target volume (PTV) was generated by adding an isotropic margin of 0.5 cm around the CTV. The organs at risk (OARs) included the heart, lungs, liver, spinal cord, and bowel bag. We selected cases in which the RGEA was within a 2 cm distance from the PTV, as in such cases it would be expected that the RGEA would receive a considerable dose of radiation. The RGEA was identified just below the pylorus in the axial CT slices and was delineated along the right part of the stomach's greater curvature. The coronal view was used for validity check (Figure 1).

All cases were planned using the volumetric modulated arc therapy (VMAT) technique, applying 3 to 4 arcs with a beam energy of 10 MV. The RGEA dosimetry was evaluated based on the following parameters: maximal dose (Dmax), mean dose (Dmean), V10Gy, and V15Gy. We sought to keep the RGEA's V10Gy and V15Gy below 15% and 10% respectively. In terms of coverage objectives, we required that D95% of PTV receive at least 95% of the prescription dose and that D2% receive no more than 107% of the prescription dose. The relative dose reductions in the above-mentioned four dose parameters were calculated. Significant relative dose reduction was defined as at least a 20% decrease after replanning was performed.

## Results

Six eligible cases were retrieved from our database; four patients had distal esophageal cancer while the remaining two had GEJ carcinoma. All patients had histologically proven adenocarcinoma of the lower esophagus/GEJ. Three patients had a primary tumor invading the muscularis propria (T2), while two patients had a tumor invading the adventitia (T3). The remaining patient did not undergo an endoscopic ultrasound; therefore, his primary tumor was not assessable. Regarding nodal status, four patients had clinically positive lymph nodes (enlarged and positron emission tomography (PET)-avid in 18-Fluoro-deoxyglucose positron emission tomography (FDG-PETCT scan) located in the gastrohepatic region, while the remaining two patients did not have evidence of lymph node metastasis.

Mean CTV and PTV were 376 cm^3^ (range 302-420 cm^3^) and 663 cm^3^ (range 528-934 cm^3^) respectively. The mean distance between PTV and the RGEA was 0.78 cm (range 0-1.8 cm). Overall, we achieved a significant relative dose reduction in 23 out of 24 dosimetric parameters without compromising coverage objectives. The dosimetric values for all cases prior to and after replanning for RGEA constraints are presented in Table 1.

**Table 1:** The dosimetric data of RGEA prior (left columns) and after (right columns) replanning RGEA: Right gastroepiploic artery Coverage parameters of PTV in terms of D95% and D2% are also presented.

Case	RGEA Dmax (Gy)		RGEA V10Gy (%)		RGEA V15Gy (%)		RGEA Mean dose (Gy)		PTV D95% (%)		PTV D2% (%)	
1	31	29	21.42	11.54	13.44	5.4	8.38	5.11	96.9	97.2	103.3	103.7
2	17.56	12.52	22.66	0.2	3.6	0	5.36	2.73	97.6	96	104	105
3	18.33	7.5	14.96	0	5.5	0	5.78	1.83	98.9	98.7	103.4	103.6
4	22.86	12.68	94.72	1.73	58.59	0	15.54	7.35	95	95	104	105
5	23.72	11.65	95	1.25	81.5	0	18.36	5.63	97.4	97	103	103
6	23.26	15.6	81.9	6.88	24.5	0	13.27	5.5	97	97	103	103

**Figure 2:**
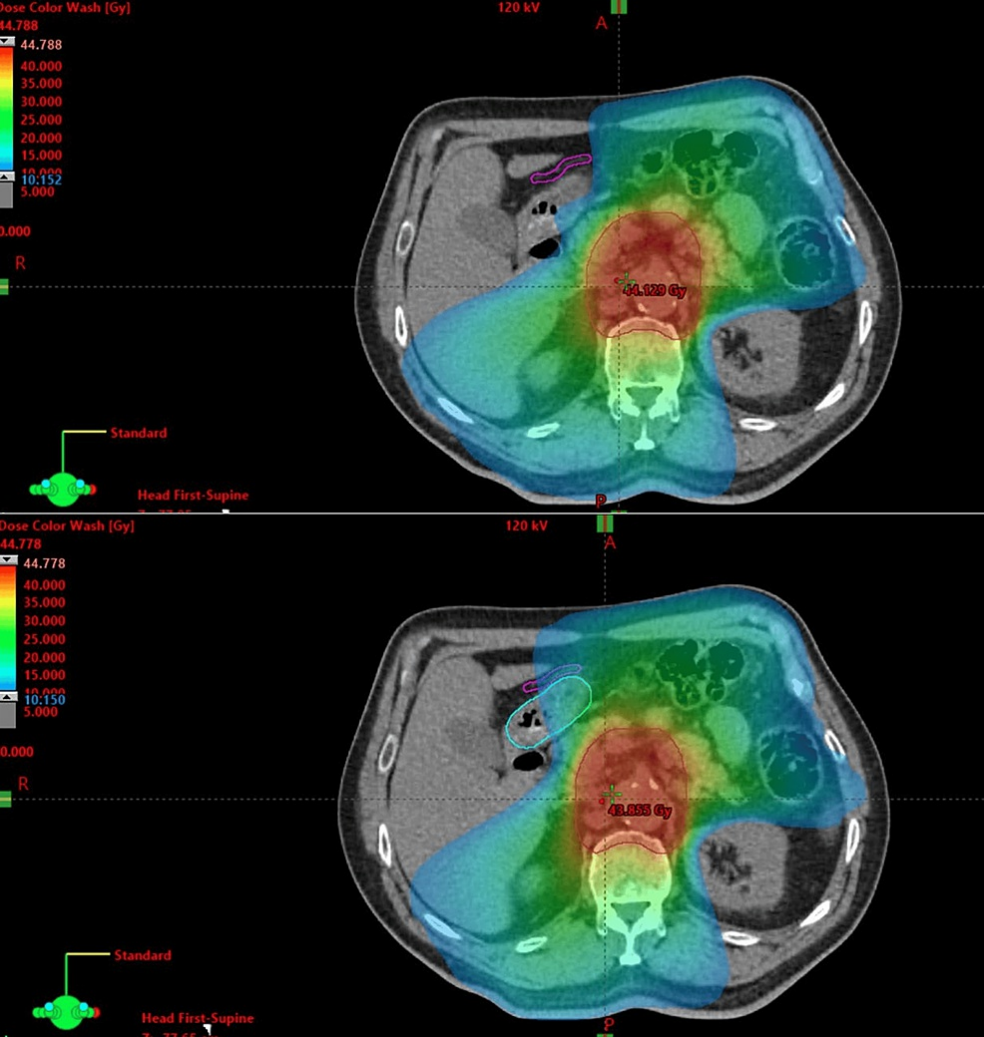
Representative axial images before (upper image) and after (lower image) replanning was performed The right gastroepiploic artery (RGEA) (purple structure) could be significantly spared from dose levels of 10 Gy and above.

**Figure 3:**
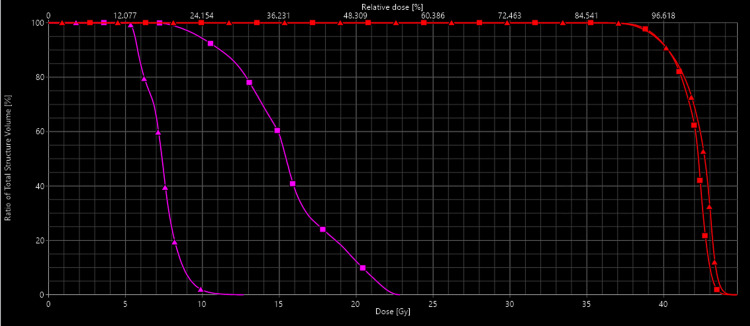
Dose-volume histogram The histogram is showing a significant dose reduction for the RGEA after replanning (purple) without compromising PTV coverage (red).

The mean relative dose reductions for V10Gy, V15Gy, Dmean, and Dmax were 88%, 93%, 49%, and 38% respectively. All OAR constraints were also met in all cases after replanning. A representative case prior to and after replanning is demonstrated in Figure 2. The corresponding dose-volume histogram is also shown in Figure 3.

## Discussion

The treatment paradigm of locally advanced esophageal/GEJ cancer is constantly evolving. Currently, the two most widely practiced strategies in this setting are perioperative chemotherapy [11] or neoadjuvant chemoradiation followed by adjuvant checkpoint inhibitor after surgery if patient did not achieve complete pathological response [12]. In either approach, it is common that patients finish the preoperative treatment phase in a poorer condition compared to baseline due to treatment-related toxicity. In particular, patients may become undernourished and hypoalbuminemic hence rendering them high-risk patients for anastomotic leaks and conduit failures. Therefore, trying to mitigate other risk factors for such complications would be prudent.

Radiation-induced endothelial damage is a well-described phenomena that could occur in relatively low radiation dose. Impaired vasomotor response, increased expression of adhesion molecules, generating a prothrombotic state, and increased cytokines levels are among the possible mechanisms of radiation-induced damage of the vascular endothelium [8]. In this study, we sought to identify and contour the RGEA, a potential critical structure that may influence postoperative morbidity rates. We endeavoured to lower the radiation doses to this vessel without compromising target coverage. In our proof-of-concept study we demonstrated that the RGEA could be identified and, subsequently delineated, relatively easy. In addition, replanning was feasible with significant radiation dose reduction to the RGEA.

Several previous studies have examined the association between various dosimetric parameters of neoadjuvant chemoradiation in esophageal cancer and postoperative morbidities. Wang et al. have shown that postoperative pulmonary complications were associated with radiation dose to the lungs [13]. However, radiation dose to the lungs could be lowered to a limited extent as many tumors stretch to a considerable length with a subsequent significant increase of the clinical target volume. This cranio-caudal length would inevitably lead to a significant exposure of the lungs to the low-dose bath and to an increase in lungs’ mean dose. The radiation dose to the gastric fundus was also studied as a predictor for anastomotic complications though the results were not consistent across studies with some demonstrating a significant association while others not [14-16]. A large retrospective study conducted by Juloori et al. showed that anastomotic leaks were more likely to occur if the anastomosis is constructed within the preoperative radiation field [14]. Again, as is the case with radiation dose to the lungs, in many cases the surgeons would have limited options in terms of the site of anastomotic construction relatively to the radiation field, i.e. this would be a predictive, rather than a remediable factor.

When pursuing neoadjuvant chemoradiation for distal esophageal and “true” GEJ carcinomas, the RGEA would most frequently be in a reasonable distance from the target volumes. Consequently, limiting the radiation dose to this vital vessel would often be feasible without compromising proper target volume coverage, as we demonstrated. Our study is a purely feasibility study from a dosimetric perspective. It lacks a clinical endpoint which is, no doubt, a significant limitation. Our ability to access the medical records of our patients after they finish CRT is limited as all of them are referred to larger centres for esophagectomy. 

## Conclusions

We propose that the RGEA should be regarded as a potential OAR that may influence the postoperative course of patients who undergo neoadjuvant CRT and surgery for esophageal/GEJ carcinomas. We demonstrated that improved dosimetry for this structure is fairly feasible without compromising dose coverage. Further studies with clinical endpoints are needed to validate an association between RGEA dosimetry and postoperative anastomosis-related sequelae.
